# Role of Interleukin-17A on the Chemotactic Responses to CCL7 in a Murine Allergic Rhinitis Model

**DOI:** 10.1371/journal.pone.0169353

**Published:** 2017-01-03

**Authors:** Yu-Lian Zhang, Doo Hee Han, Dong-Young Kim, Chul Hee Lee, Chae-Seo Rhee

**Affiliations:** 1 Department of Otorhinolaryngology-head and Neck Surgery, Seoul National University College of Medicine, Seoul, Korea; 2 Research Center for Sensory Organs, and Institute of Allergy, Seoul, Korea; 3 Department of Otorhinolaryngology-Head and Neck Surgery, Seoul National University Bundang Hospital, Seongnam, Gyeonggido; 4 Clinical Immunology, Seoul National University Medical Research Center, Seoul, Korea; Centre National de la Recherche Scientifique, FRANCE

## Abstract

**Background:**

The proinflammatory cytokine interleukin (IL)-17A is associated with eosinophil infiltration into the nasal mucosa in a mouse model of ovalbumin-induced allergic rhinitis. Chemotaxis of eosinophils is mediated primarily through C-C chemokine receptor type 3 (CCR3). However, the mechanism underlying the IL-17A-mediated enhancement of eosinophil recruitment via chemoattractants/chemokines remains unknown.

**Objectives:**

In this study, we assessed the contribution of IL-17A to eosinophil-related inflammation via the CCL7/CCR3 pathway in experimental allergic rhinitis.

**Methods:**

IL-17A knockout (KO) and wild-type (WT) BALB/c mice were injected intraperitoneally and challenged intranasally with OVA to induce allergic rhinitis. Various parameters of the allergic response were evaluated, and mRNA and protein levels of CCL7 and CCR3 in nasal tissue and serum were compared between the two groups. The chemotactic response to CCL7 with or without IL-17A in bone marrow-derived eosinophils (bmEos) from BALB/c mice was measured.

**Results:**

In the allergic rhinitis model, IL-17A deficiency significantly decreased nasal symptoms, serum IgE levels, and eosinophil recruitment to the nasal mucosa. CCL7 and CCR3 mRNA and protein levels were decreased in the nasal mucosa of IL-17A KO mice compared with the WT mice. BmEos showed a significantly increased chemotactic response to -low concentration of CCL7 in the presence of IL-17A compared with its absence.

**Conclusion:**

The suppression of nasal inflammation due of IL-17A deficiency in allergic rhinitis is partly responsible for the regulation of CCL7 secretion and eosinophil infiltration, which may be regulated via the CCL7/CCR3 pathway.

## Introduction

Allergic rhinitis is a typical Th2 cytokine-dominant disease that involves the influx of numerous inflammatory cells, such as eosinophils and mast cells, into the nasal mucosa as well as elevated immunoglobulin E (IgE) production [1, 2]. Th17 cells are developmentally different from Th1 and Th2 cells and primarily produce interleukin (IL)-17 [3]. IL-17A may represent a link between the activation of T-lymphocytes and the mobilization of neutrophils [4, 5]. However, few studies have examined the involvement of IL-17A in eosinophil infiltration at sites of inflammation. Th17 cells enhance eosinophilic airway inflammation, which is mediated by Th2 cells [6]. In nasal polyps, IL-17A plays an important role in eosinophil accumulation and in subepithelial layers; its sites of expression largely coincide with eosinophils and CD4^+^ lymphocytes, but not with neutrophils [7].

C-C chemokine receptor type 3 (CCR3) is involved in the development of allergic diseases such as allergic rhinitis, asthma and atopic dermatitis [8]. It is expressed on eosinophils, mast cells, basophils, neutrophils, and endothelial cells; among these, it is most highly expressed on eosinophils [9]. CCR3 is responsible for eosinophil chemotaxis toward eotaxin, regulated upon activation, normal T-cell expressed and secreted (RANTES), and CCL7 [9]. CCL7 is a small chemokine [10, 11] secreted by various cells, including macrophages, monocytes, mast cells, epithelial cells, fibroblasts, and endothelial cells. After its secretion, CCL7 binds to the main receptors CCR1, CCR2, and CCR3 on chemotactic eosinophils, monocytes, basophils, T lymphocytes, neutrophils, and natural killer cells [12]. CCL7 mRNA levels in the bronchial mucosa and CCL7 protein levels in bronchoalveolar lavage fluid were significantly increased in both atopic and non-atopic asthmatic patients; moreover, CCL7 was shown to cause eosinophil accumulation in the bronchial mucosa [13, 14]. Allergic rhinitis is associated with elevated expression of CCL7, which may be closely associated with the recruitment of inflammatory cells [15]. Several reports have addressed the relationship between IL-17A and CCL7 in mouse embryonic fibroblasts and MLE12 cells (a mouse lung epithelial cell line), brain and central nervous system tissues, and models of chronic asthma [16, 17]. However, the exact relationship among CCL7, eosinophils, and IL-17A in allergic rhinitis in the upper airway remains unclear.

Recent studies using a mouse model of asthma showed that IL-17A induces the recruitment of not only neutrophils, but also eosinophils, into the airway [6, 18]. Additionally, IL-17A deficiency attenuated allergic inflammation in a mouse model of allergic rhinitis [19]. However, the mechanism behind IL-17A-induced suppression of eosinophil inflammation remains unknown. Thus, in this study, we investigated the regulation of eosinophil inflammation by IL-17A and the involvement of the CCL7/CCR3 pathway in a mouse model of allergic rhinitis.

## Materials and Methods

### Animals

Four-week-old female wild-type (WT) BALB/c mice (Orient, Gyeonggi, South Korea) and IL-17A knockout (KO) mice (18~20 g) were used in all experiments. IL-17A KO mice with a BALB/c background were obtained from Prof. Yoichiro Iwakura (Center for Experimental Medicine, Institute of Medical Science University of Tokyo, Japan) [20]. Animal experiments were approved by the Institutional Animal Care and Use Committee of Seoul National University, South Korea (IACUC number: 11–0325). Mice were maintained under specific-pathogen-free conditions. All mice were housed in a temperature controlled environment with a 12 hour dark/light cycle. During the experimental period (4 weeks), the weight of each mouse was measured every week at the same time. Mice were euthanized at the end of a study by isoflurane chamber. There are no mice without euthanasia.

### Ovalbumin sensitization and nasal challenge

**Fig 1:**
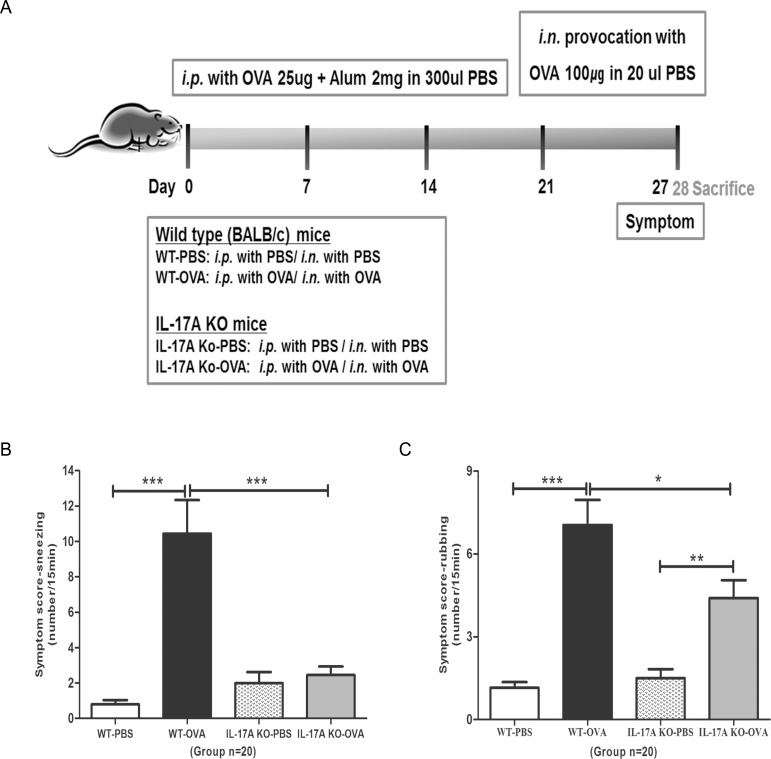
Experimental protocol and nasal symptoms. (A) Mice were sensitized by intraperitoneal injection of ovalbumin [28] mixed with aluminum hydroxide on days 0, 7, and 14. Daily OVA intranasal challenge was performed from days 21 to 27. The frequencies of (B) sneezing and (C) rubbing events were counted over a 15-min period after OVA provocation. Results are means ± SEM (*n* = 20). (*** *P* < 0.001, ** *P* < 0.01, * *P* < 0.05).

The mice were divided into four groups. The negative control group (WT-PBS) was sensitized and challenged with phosphate-buffered saline (PBS), the positive control group (WT-OVA) was sensitized and challenged with OVA, the IL-17A KO-PBS group consisted of IL-17A KO mice sensitized and challenged with PBS, and the IL-17A KO-OVA group consisted of IL-17A KO mice sensitized and challenged with OVA. The schedule for allergen sensitization and intranasal challenge is summarized in Fig 1A. Briefly, the WT-OVA and IL-17A KO-OVA groups were sensitized by intraperitoneal injection of 25 μg OVA mixed with 2 mg alum on days 0, 7, and 14 and then challenged by intranasal treatment of 100 μg OVA for 7 consecutive days, from days 21 to day 27. The WT-PBS and IL-17A KO-PBS groups were injected intraperitoneally and challenged intranasally with PBS following the same schedule.

### Evaluation of nasal symptoms

On day 27, after the final administration of OVA, the frequencies of sneezing and nose scratching were counted for 15 min by a blinded observer to evaluate early allergic responses.

### Serum levels of total and OVA-specific IgE

Serum samples were stored at -70°C before using for measurements of total IgE and OVA-specific IgE, as described previously [19]. Briefly, total serum IgE was measured by a standard enzyme-linked immunosorbent assay (ELISA) using an anti-mouse IgE capture monoclonal antibody (BD Pharmingen, San Diego, CA, USA) and horseradish peroxidase (HRP)-conjugated anti-mouse IgE (Southern Biotechnology, Birmingham, AL, USA). To detect OVA-specific IgE, 96-well immune plates were coated with 100 ug/mL of OVA in carbonate-bicarbonate buffer. After the serum samples had been incubated for 2 h, biotin-conjugated rat anti-mouse IgE monoclonal antibody (BD Pharmingen) and streptavidin-HRP (BD Pharmingen) were used to detect OVA-specific IgE levels.

### Histological analysis

The heads of mice were fixed in 10% formalin, decalcified, and embedded in paraffin wax. Nasal tissues were sectioned and stained with hematoxylin and eosin (H&E) for inflammatory cell counting and with Sirius red for eosinophil counting. For neutrophils staining, sections were immunostained with the rat anti-mouse neutrophil antibody NIMP-R14 (Abcam, Cambridge, MA, USA) and subsequently with anti-rat IgG (Dako, Copenhagen, Denmark). Bound antibodies were visualized by 3.3’-diaminobenzidine (DAB) kit (Vector Laboratories, Burlingame, CA, USA).

### Quantitative polymerase chain reaction (qPCR)

Cells were prepared from the nasal mucosa and lysed in TRIzol reagent (Invitrogen, Carlsbad, CA, USA). cDNA was synthesized using Superscript Reverse Transcriptase II (Invitrogen) and oligo-(dT) primers (Thermo Fisher Scientific, Burlington, VT, USA). Primers ad probes specific to IL-17A (Mm00439618_m1), IL-4 (Mm00445258_g1), IL-5 (Mm00439646_m1), IL-10 (Mm00439616_m1), interferon (IFN)-γ (Mm99999071_m1), CCL7 (Mm00443113_m1), CCR3 (Mm01216172-m1), and glyceraldehyde 3-phosphate dehydrogenase (GAPDH) (Mm03302249_g1), were purchased from Applied Biosystems (Foster City, CA, USA). The reaction was assayed using the ABI PRISM 7000 sequence detection system (Applied Biosystems). Expression levels of the target genes were normalized to those of GAPDH.

### CCL7 secretion in nasal lavage fluid (NLF) and serum

After sacrifice, NLF was obtained from the upper airway by rinsing twice with 200 μL PBS. NLF and serum concentrations of CCL7 were measured using CCL7 Instant ELISA kits (eBiosience, Vienna, Austria) and CCL7 Development ELISA kits (PeproTech, Rocky Hill, NJ, USA), respectively. The minimal detection limits for these kits are 2.6 and 8 pg/mL, respectively.

### Western blot analysis of CCR3 in the nasal mucosa

Proteins were obtained from the nasal mucosa using lysis buffer (Sigma, St Louis, MO, USA). Proteins (50 μg) were separated on 8%-16% Tris-glycine mini gels (Invitrogen, San Diego, CA, USA). Western blotting of CCR3 and β-actin was performed using a primary rabbit monoclonal anti-CCR3 antibody (Abcam, Cambridge, UK) and anti-β-actin antibody (Cell Signaling, Danvers, MA, USA), respectively. Quantification of Western blot bands was performed using TINA 2.0 software.

### Flow cytometric assessment of blood cells

BALB/c WT and IL-17A KO mice were sensitized and exposed to PBS or OVA. Whole blood was collected in tubes containing heparin. Packed whole blood (50 μL) was stained with V450-anti-CD45, phycoerythrin (PE)-anti-Siglec-F and adenomatous polyposis coli (APC)-anti-CCR3 to determine the proportion of eosinophils among blood cells. Data were acquired using the BD LSR II flow cytometer (BD Biosciences, San Jose, CA, USA) and analyzed using FlowJo software (Treestar).

### Isolation and detection of bone marrow-derived eosinophils (bmEos)

BmEos were generated as described elsewhere [21]. Briefly, bone marrow cells were flushed from the femora and tibiae of 4-week-old BALB/c mice and cultured in RPMI-1640 medium (Welgene, Gyeongsan, South Korea) containing 20% fetal bovine serum, 100 IU/mL penicillin, 10 ug/mL streptomycin, 2 mM glutamine, 25 mM HEPES, 1 × nonessential amino acids, 1 mM sodium pyruvate (Life Technologies, Grand Island, NY, USA), 50 μM β-mercaptoethanol (Amresco, Solon, OH, USA) and supplemented with 100 ng/mL stem-cell factor (PeproTech) and 100 ng/mL FLT3-Ligand (PeproTech) from days 0 to 4. From day 4, cell medium was replaced with medium containing only 10 ng/mL IL-5 (PeproTech). On day 14, bmEos were incubated with PE-conjugated rat anti-mouse Siglec-F (BD Biosciences) and APC-conjugated anti-mouse CCR3 (R&D Systems, Minneapolis, MN, USA). Data were acquired using the FACSCalibur flow cytometer (BD Biosciences) and analyzed using Cell Quest Prosoftware (ver. 5.2.1 BD Biosciences). Siglec-F and CCR3-positive cells were identified by comparison with PE-conjugated IgG2a, κ isotype, and APC-conjugated IgG2a isotype controls.

### Chemotaxis assay

A chemotaxis assay was performed in Transwell plates (Corning, Corning, NY, USA) using a 5.0-μm pore size polycarbonate membrane. Various concentrations (0.1, 1 and 10 ng/mL; R&D Systems) of murine IL-17A and 100 or 500 ng/mL of murine CCL7 (Peprotech) proteins were diluted in 0.6ml of medium containing 10 ng/ml recombinant murine IL-5 (Peprotech). The medium was placed in the lower wells of the plates, and the bmEos (100 μL, 5×10^6^ /mL) were placed in the upper chambers. Cells were incubated at 37°C to permit migration across the membrane in response to CCL7 with or without IL-17A. After 3 h, the upper chambers were removed, and the number of cells that migrated to the bottom chamber in 1 min was counted using the FACSCalibur flow cytometer.

### Statistical analysis

Results are expressed as means ± standard error mean (SEM). Statistical analysis was performed using Graphpad Prism software (version 5.0). A Mann-Whitney U-test was used to evaluate differences between mean values. A *P* value of < 0.05 was considered to indicate statistical significance.

## Results

### Symptom scores

The frequencies of nasal sneezing and rubbing in the positive control group (WT-OVA) were 10.45 ± 1.89 and 7.05 ± 0.91, respectively (Fig 1B and 1C). In comparison, these scores were significantly reduced in the IL-17A KO-OVA group (2.45 ± 0.48, *P* < 0.001, and 4.40 ± 0.65, *P* = 0.029, respectively).

### Decreased IgE levels in serum and eosinophil infiltration in the nasal mucosa

**Fig 2:**
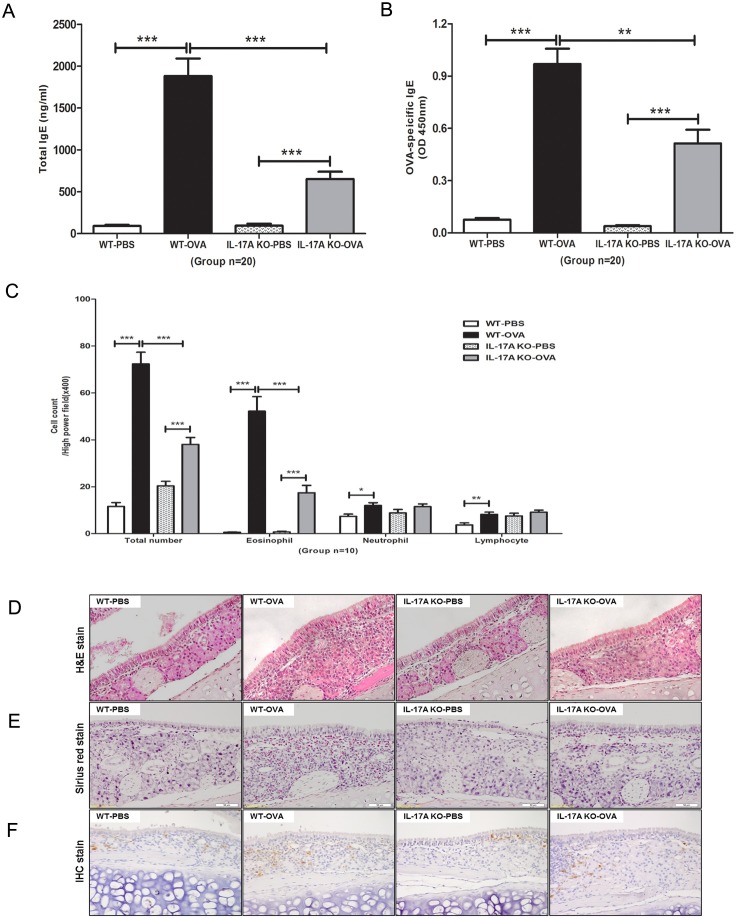
Comparison of serum immunoglobulin E (IgE) levels and eosinophilic infiltration levels. Serum levels of (A) total IgE and (B) OVA-specific IgE were significantly lower in the IL-17A KO-OVA mice than in the WT-OVA mice (*n* = 20). (C) The total inflammatory cells and eosinophils were significantly reduced (*n* = 10) in the IL-17A KO-OVA group compared with that in the WT-OVA group. Results are means ± SEM. Histological findings in the nasal mucosa in each group (×400 magnification), with (D) hematoxylin and eosin staining of lymphocytes, (E) Sirius red staining of eosinophils and (F) Immunohistochemistry staining of neutrophils. (*** *P* < 0.001, ** *P* < 0.01, * *P* < 0.05).

The serum levels of total IgE (*P* < 0.001; Fig 2A) and OVA-specific IgE levels (*P* < 0.01; Fig 2B) in the IL-17A KO-OVA group were decreased significantly compared with those in the WT-OVA group. Lymphocytes, eosinophils and neutrophils in nasal mucosal tissue were detected by H&E staining, Sirius red staining and immunohistochemistry, respectively. Total inflammation cells and eosinophils infiltration in the submucosa of the WT-OVA group was significantly higher than that in the IL-17A KO-OVA group (*P* <0.001, *P* <0.001, respectively; Fig 2C–2F).

### Cytokine mRNA expression levels in nasal mucosa

**Fig 3:**
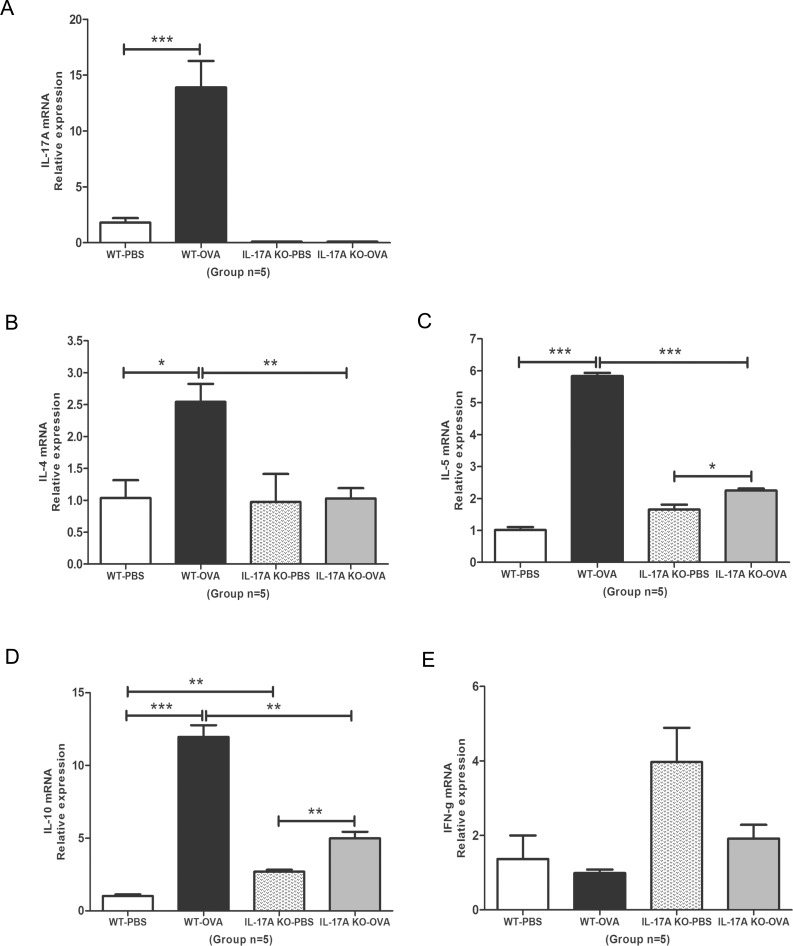
Comparison of the following cytokine mRNA levels in the nasal mucosa. (A) interleukin (IL)17A, (B) IL-4, (C) IL-5, (D) IL-10, and (E) interferon (IFN)-γ. mRNA levels of the target genes were normalized to those of glyceraldehyde 3-phosphate dehydrogenase and expressed as fold changes relative to the WT-phosphate-buffered saline group. Results are means ± SEM (*n* = 5). (*** *P* < 0.001, ** *P* < 0.01, * *P* < 0.05). Figures are representative results from at least two independent experiments.

24 hours after the last challenge with ovalbumin, IL-17A mRNA levels were measured by real time PCR. In WT-OVA groups, IL-17A mRNA expression was significantly increased in nasal mucosa compared with WT-PBS groups (P < 0.001; Fig 3A). mRNA expression of IL-4 (*P* < 0.01; Fig 3B), IL-5 (*P* < 0.001; Fig 3C) and IL-10 (*P* < 0.01; Fig 3D) in the nasal mucosa was significantly reduced in the IL-17A KO-OVA group compared with the WT-OVA group. The levels of IFN-γ mRNA did not differ significantly between the IL-17A KO-OVA and WT-OVA groups (Fig 3E). IL-10 mRNA is only increased in the IL-17A KO control group compared to WT control but not in the IL-17A KO-OVA group as stated in the first sentence of this paragraph (*P* < 0.001; Fig 3D).

### Decreased CCL7 mRNA and protein levels in IL-17A KO mice

**Fig 4:**
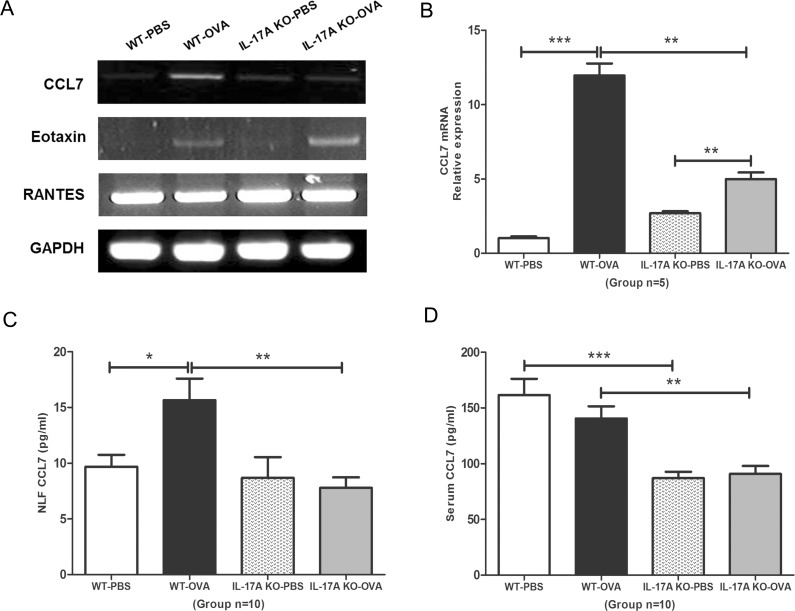
Comparison of CCL7 mRNA and protein expression levels. (A) The mRNA levels of a variety of chemokines associated with eosinophil chemotaxis, including eotaxin, RANTES, and CCL7 were measured by Reverse Transcription polymerase chain reaction (RT-PCR). (B) Quantitative polymerase chain reaction (qPCR) was performed to measure CCL7 mRNA levels (*n* = 5) in the nasal mucosa. CCL7 protein levels were measured by enzyme-linked immunosorbent assay in (C) nasal lavage fluid and (D) serum (*n* = 10). Results are means ± SEM (*** *P* < 0.001, ** *P* < 0.01, * *P* < 0.05). Fig 4A and 4B are representative results from at least two independent experiments.

We measured the mRNA expression of several chemokines, including eotaxin, RANTES, and CCL7, in the nasal mucosa. Of these, only CCL7 mRNA levels were significantly decreased in the IL-17A KO-OVA group compared with WT-OVA group (*P* < 0.01, Fig 4A and 4B). The CCL7 protein level in NLF was also significantly lower in the IL-17A KO-OVA group (7.79 ± 0.94 pg/mL) than in the WT-OVA group (15.65 ± 1.94 pg/mL, *P* < 0.01; Fig 4C). The CCL7 serum protein levels were significantly lower (86.99 ± 5.71 pg/mL) in the IL-17A KO-PBS and IL-17A KO-OVA groups (90.73 ± 7.18 pg/mL) than in the WT-PBS (161.40 ± 14.62 pg/mL, *P* < 0.001) and WT-OVA groups (140.40 ± 10.93 pg/mL, *P* < 0.01), respectively (Fig 4D).

### Decreased CCR3 mRNA and protein levels in the nasal mucosa of IL-17A KO mice

**Fig 5:**
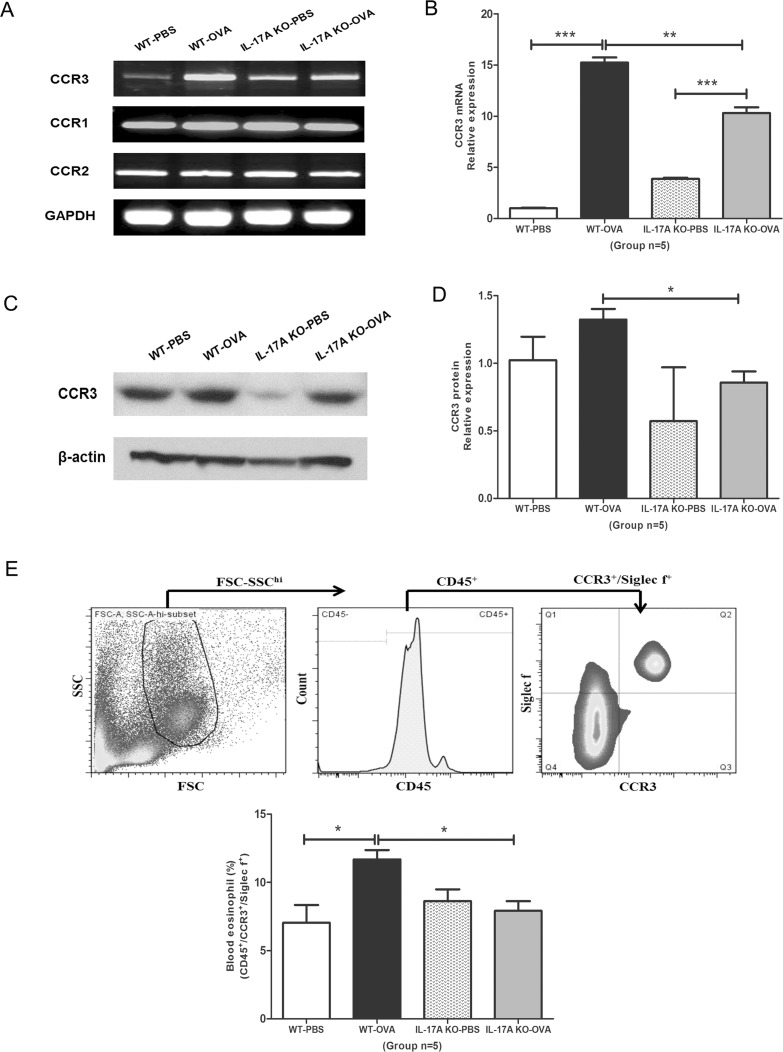
Comparison of CCR3 mRNA and protein levels. (A) mRNA levels of the CCL7 receptors CCR1, CCR2, and CCR3 were measured by RT-PCR. (B) mRNA levels of CCR3 (*n* = 5) in the nasal mucosa were measured by qPCR. (C, D) The protein levels of CCR3 in the nasal mucosa were measured by (*n* = 5) Western blot analysis. (E) Flow cytometric analysis of CCR3 expression in eosinophils in blood. Results are means ± SEM (*n* = 5) (*** *P* < 0.001, ** *P* < 0.01, * *P* < 0.05). Figures are representative results from at least two independent experiments.

CCL7 interacts with three receptors: CCR1, CCR2, and CCR3. Among these, the mRNA level of only CCR3 (*P* < 0.001) was significantly decreased in the IL-17A KO-OVA group compared with WT-OVA group (Fig 5A and 5B). The CCR3 protein level was measured in the nasal mucosa by Western blotting and was also significantly decreased in the IL-17A KO-OVA group compared with WT-OVA group (P < 0.05; Fig 5C and 5D).

### Decreased CCR3 expression levels in blood eosinophils in IL-17A KO-OVA mice

Blood samples from the WT-OVA and IL-17A KO-OVA mice were stained with anti-CD45, anti-Siglec-F, and anti-CCR3 antibodies to detect CCR3 expression in eosinophils. The CCR3expression of eosinophils in blood was lower in IL-17A KO-OVA mice than in WT-OVA mice (11.68 ± 0.69% vs. 7.92 ± 0.70%, respectively; *P* = 0.016; Fig 5E).

### Increased CCL7 chemotactic activity to bmEos in the presence of IL-17A

**Fig 6:**
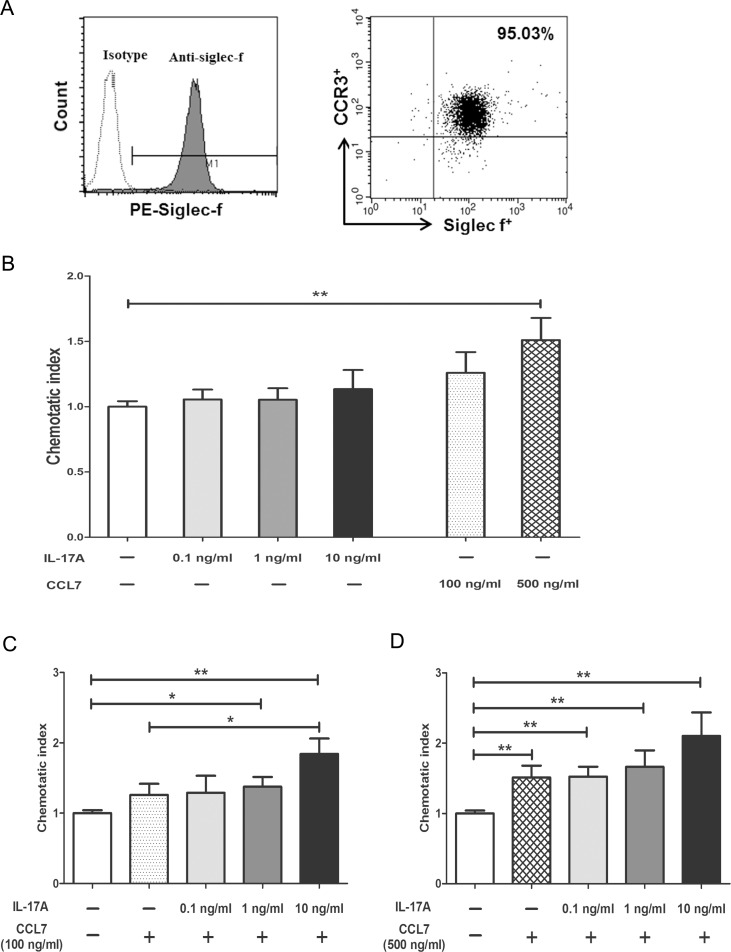
Comparison of chemotactic responses in bone marrow-derived eosinophils (bmEos). (A) BmEos were cultured until a high-purity (>95%) was reached. (B-D) BmEos were suspended and placed on the upper chambers, in the presence or absence of CCL7 and/or IL-17A, kept in the bottom chamber. BmEos without IL-17A and CCL7 proteins were used as the control. The bmEos exhibit chemotaxis to (B) 100 or 500 ng/mL CCL7 and various concentrations (0.1, 1 and 10 ng/mL) of IL-17A protein, respectively. (C) 100 ng/ml of CCL7 coupled with IL-17A (0.1, 1 and 10 ng/mL), (D) 500 ng/ml of CCL7 coupled with IL-17A (0.1, 1 and 10 ng/mL). Results are means ± SEM (*n* = 4). (** *P* < 0.01, * *P* < 0.05).

BmEos were cultured for 14 days until a high purity (>95%) was reached (Fig 6A). There were no differences in CCR3 expression in eosinophils induced from the bone marrow of naïve IL-17A KO mice and WT mice in our experiment (S1 Fig). In this transwell chemotaxis assay, demonstrated chemotactic responses of bmEos to 100 and 500 ng/mL CCL7 and to Various concentrations (0.1, 1 and 10 ng/mL) of IL-17A protein (Fig 6B–6D). BmEos without IL-17A and CCL7 proteins were used as the control. When CCL7 protein was not added, IL-17A did not have chemotactic effects on bmEos at all tested concentrations (0.1, 1, and 10 ng/mL) (Fig 6B). Although CCL7 increased bmEos chemotaxis compared with that of the control, the difference was only significant for high concentrations (500 ng/ml) of CCL7 (*P* < 0.01; Fig 6B). However, adding 1 or 10 ng/mL IL-17A significantly increased the capacity of low-concentration (100 ng/ml) CCL7 to induce bmEos chemotaxis compared with the control (*P* < 0.05, *P* < 0.01, respectively; Fig 6C). In addition, coupling 10 ng/mL of IL-17A with 100 ng/mL of CCL7 significantly increased the migration of bmEos compared with that of cells treated with 100 ng/mL CCL7 alone (*P* < 0.05; Fig 6C). A high concentration (500 ng/mL) of CCL7 significantly increased the chemotactic responses of bmEos compared with the control when coupled with interleukin-17A (*P* < 0.01, *P* < 0.01, *P* < 0.01, *P* < 0.01, respectively; Fig 6D).

## Discussion

This study was undertaken to determine whether eosinophil migration to the nasal mucosa in response to IL-17A is regulated by the CCL7/CCR3 pathway in a mouse model of allergic rhinitis.

According to the existing literature, IL-17A is detected in the cytoplasm of epithelial cells from the nasal mucosa of OVA-treated mice. Moreover, the expression of IL-17A mRNA level is also increased [22, 23]. These findings are consistent with our results that IL-17A mRNA level was markedly higher in the WT-OVA group than in the IL-17A KO-OVA group. IL-17A induces allergen-specific Th2 cell activation, eosinophil accumulation, and serum IgE production [18, 20]. Consistent with other studies, our results in an allergic rhinitis mouse model confirmed that IL-17A deficiency is associated with decreased serum IgE production and IL-4 expression in nasal tissue. Thus, IL-17A deficiency may play an important role in the development of allergic rhinitis by reducing Th2 cytokine levels. Results of a 2015 report on an IL-17A-KO ovalbumin-induced allergic rhinitis model were inconsistent with our current findings [23]. In our present experiment, we used BALB/c mice, which are known as the best model for allergic rhinitis induced with ovalbumin, whereas in the 2015 report, the authors used C57BL/6J mice. Our model showed more consistent results, with considerably higher expression of IL-17A mRNA as well as higher levels of inflammatory factor in the nasal mucosa. Hence, we speculate that the heterogeneity of the mice may underlie the differences in results.

We also observed different local responses with respect to IFN-γ and IL-10 in the IL-17A KO mice; in particular, the basal IL-10 level in the nasal mucosa was significantly increased in the IL-17A KO mice compared with the WT mice. These findings may attribute to negative regulation of cytokine production in regulatory T cells by IL-17A [18]. Although this did not reach statistical significance, the expression of IFN-γ mRNA level was elevated in the IL-17A KO-PBS group. This result can be partially explained by the previous report that the IL-17A have protective function in the wasting disease by suppresses Th1 differentiation [24]. However, further studies are needed to determine the exact mechanisms involved.

In allergic diseases, IL-5 is a key cytokine involved in the induction of eosinophil recruitment [25]. IL-5 is necessary for long-term eosinophilia, and is the major factor inducing eosinophil differentiation from lineage-committed precursors, after which it extends the lifespan of infiltrating eosinophils [26]. IL-17A is a critical cytokine in a number of inflammatory diseases. A previous report suggested that IL-17A has the potential to promote airway inflammatory cell infiltration [27]. In addition, in a model of chronic eosinophilia, IL-17A KO mice failed to accumulate the critical number of eosinophils after allergen challenge [28]. This is consistent with our results obtained from a model of allergic rhinitis: the infiltrating eosinophil number and IL-5 mRNA levels in nasal mucosa were significantly decreased in the IL-17A KO-OVA group compared with the WT-OVA group.

In the present study, CCL7 and CCR3 mRNA levels were significantly increased in the nasal mucosa in an allergic rhinitis. This is consistent with previous reports of greater than 90% upregulation of CCL7 and CCR3 gene expression in the nasal mucosa of patients with allergic rhinitis [29]. In addition, in a previous study, IL-17 transgene overexpression induced inflammation in the lungs, CCL7 expression was significantly increased and IL-17-treated MLE12 cells showed a similar gene expression [17]. In our study, significantly reduced CCL7 mRNA and protein levels were found in the nasal mucosa and NLF in the IL-17A KO-OVA group compared with the WT-OVA group. The serum CCL7 level was also significantly lower in the IL-17A KO mice. Our results in nasal tissues are similar to those in other tissues observed in previous studies. Interestingly, the WT-PBS group had mildly higher CCL7 secretion than the WT-OVA group in the serum, but the difference was not significant. IL-4, IL-10, and IL-13 have been reported to suppress CCL7 expression in endothelial cells and monocytes [30]. Thus, we predict that CCL7 secretion may be mildly decreased in the WT-OVA group compared with that in the WT-PBS group. However, further studies would be required to substantiate this.

CCR3 is expressed by eosinophils, basophils, mast cells, neutrophils, Th2 cells, and endothelial cells. Among these, eosinophils exhibit the highest CCR3 expression levels [12, 31, 32]. Siglec-F has emerged as a reliable marker for detecting eosinophils in mice [33]. In the present study, we compared CCR3 expression in eosinophils in blood samples obtained from the OVA challenged WT and IL-17A KO mice. Eosinophil CCR3 expression was decreased in the IL-17A KO mouse group. This suggests that IL-17A deficiency reduces CCR3 expression in eosinophils in blood.

In this study, we used an allergic rhinitis mouse model and found that IL-17A deficiency reduced eosinophils important to allergic rhinitis. We focused on the effects of CCR3, which are expressed by eosinophils and its chemokine CCL7. Hence, we have not yet examined the differences in Th1, Th2, Th17, and regulatory T cell differentiation between the WT group and IL-17A KO group. We need further studies, including T cell differentiation studies for evaluating possible mechanism.

CCR3 expression on eosinophils is responsible for chemotaxis by eotaxin, RANTES, and CCL7 [9, 34]. In this study, IL-17A deficiency suppressed only the mRNA level of CCL7, but not eotaxin or RANTES. The results of the present study correspond to those of earlier reports in that IL-17A did not induce mRNA expression of eotaxin or RANTES in bronchial epithelial cells [35]. In addition, in this study, the protein levels of CCL7 in NLF and serum were decreased in IL-17A KO-OVA group. CCR3 showed an expression profile similar to that of CCL7 in our study, and the number of eosinophils expressing CCR3 was decreased in the nasal mucosa and blood of IL-17A KO-OVA mice. The present study demonstrated that a high concentration of CCL7 independently induced chemotaxis of eosinophils, consistent with the findings of previous studies [36]. However, a low concentration of CCL7 was found to significantly increase chemotactic responses of eosinophils only when coupled with IL-17A. Based on these findings, we speculated that IL-17A may play a role in the process of low concentration of CCL7-induced eosinophil chemotaxis. Consequently these results suggest that IL-17A regulates eosinophil inflammation via the CCL7/CCR3 pathway in a mouse model of allergic rhinitis.

## Conclusions

IL-17A deficiency suppressed eosinophil infiltration and reduced symptoms by controlling early- and late- phase responses in an animal model of allergic rhinitis. IL-17A deficiency resulted in decreased mRNA and protein levels of CCL7 and CCR3. CCL7 along with IL-17A significantly elevated the chemotactic response of eosinophils. These results suggest that IL-17A plays a role in the development of allergic rhinitis by regulating the levels of Th2 cytokines, chemokines, and chemokine receptors in eosinophils. In conclusion, decreased eosinophil infiltration by IL-17A deficiency in allergic rhinitis is attributed at least in part to a decreased chemotactic response of eosinophils via the CCL7/CCR3 pathway.

## Supporting Information

S1 Fig Comparison of CCR3 expression in bmEos between WT and IL-17A KO mice. (TIF)

## Abbreviations

BmEos: Bone marrow-derived eosinophils
CCL7: Chemokine (C-C motif) ligand 7
CCR3: C-C chemokine receptor type 3
IL-17: Interleukin-17
IL-17A KO-PBS: IL-17A knockout mice sensitized and challenged with PBS
IL-17A KO-OVA: IL-17A knockout mice sensitized and challenged with OVA
OVA: Ovalbumin
WT-PBS: Wild type mice sensitized and challenged with PBS
WT-OVA: Wild type mice sensitized and challenged with OVA
