# Supplementary figures and images for: Role of Interleukin-17A on the Chemotactic Responses to CCL7 in a Murine Allergic Rhinitis Model
Source: PLoS One. 2017 Jan 3;12(1):e0169353. doi: 10.1371/journal.pone.0169353 (PMC5207516; doi:10.1371/journal.pone.0169353)

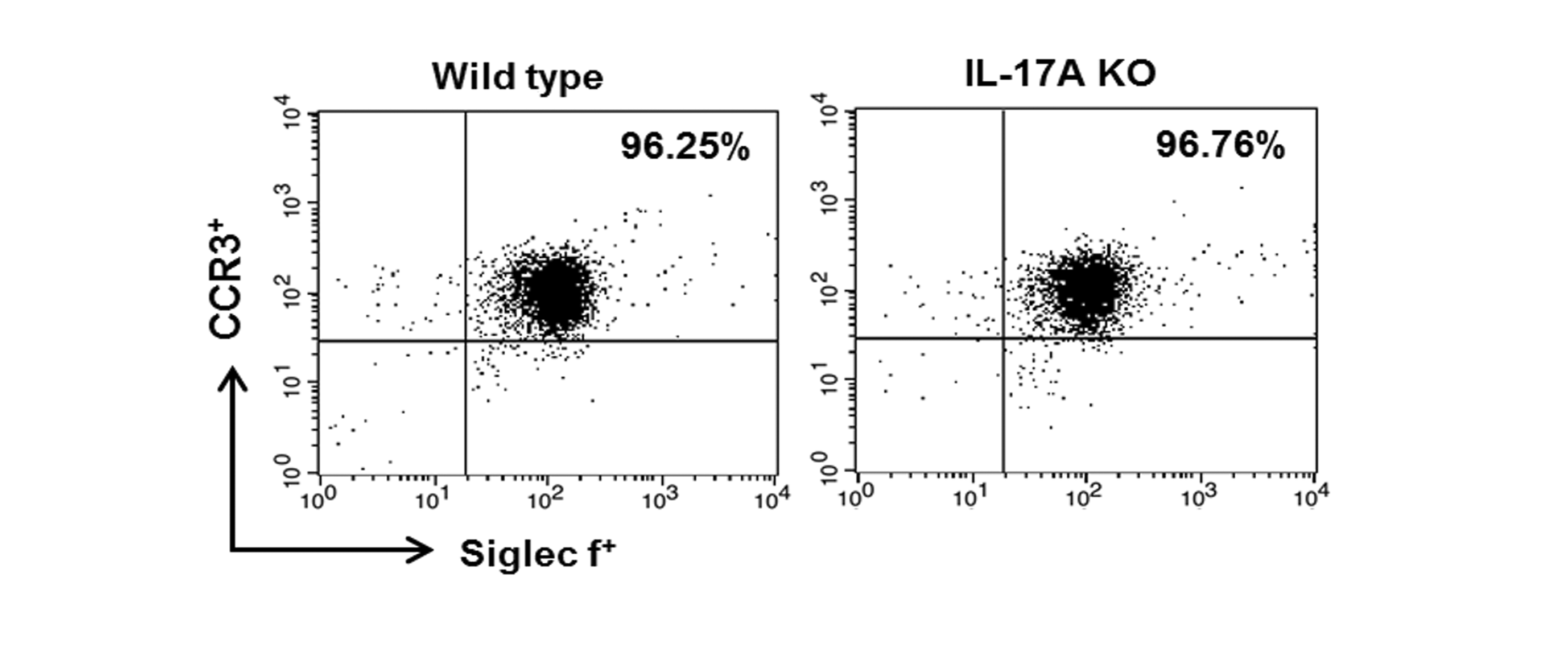

Supplement: S1 Fig — (TIF) [file pone.0169353.s001.tif]
